# Transcriptional Profiling of Hydrogen Production Metabolism of *Rhodobacter capsulatus* under Temperature Stress by Microarray Analysis

**DOI:** 10.3390/ijms160613781

**Published:** 2015-06-16

**Authors:** Muazzez Gürgan, Nilüfer Afşar Erkal, Ebru Özgür, Ufuk Gündüz, Inci Eroglu, Meral Yücel

**Affiliations:** 1 Department of Biological Sciences, Middle East Technical University, Ankara 06800, Turkey; E-Mails: muazzez@metu.edu.tr (M.G.); niluferafsar@gmail.com (N.A.E.); ufukg@metu.edu.tr (U.G.); 2 Micro-Electro-Mechanical Systems, Middle East Technical University, Ankara 06800, Turkey; E-Mail: bebru@metu.edu.tr; 3 Department of Chemical Engineering, Middle East Technical University, Ankara 06800, Turkey; E-Mail: ieroglu@metu.edu.tr

**Keywords:** *Rhodobacter capsulatus*, microarray analysis, gene expression, heat and cold stress

## Abstract

Biohydrogen is a clean and renewable form of hydrogen, which can be produced by photosynthetic bacteria in outdoor large-scale photobioreactors using sunlight. In this study, the transcriptional response of *Rhodobacter capsulatus* to cold (4 °C) and heat (42 °C) stress was studied using microarrays. Bacteria were grown in 30/2 acetate/glutamate medium at 30 °C for 48 h under continuous illumination. Then, cold and heat stresses were applied for two and six hours. Growth and hydrogen production were impaired under both stress conditions. Microarray chips for *R. capsulatus* were custom designed by Affymetrix (GeneChip^®^. TR_RCH2a520699F). The numbers of significantly changed genes were 328 and 293 out of 3685 genes under cold and heat stress, respectively. Our results indicate that temperature stress greatly affects the hydrogen production metabolisms of *R. capsulatus*. Specifically, the expression of genes that participate in nitrogen metabolism, photosynthesis and the electron transport system were induced by cold stress, while decreased by heat stress. Heat stress also resulted in down regulation of genes related to cell envelope, transporter and binding proteins. Transcriptome analysis and physiological results were consistent with each other. The results presented here may aid clarification of the genetic mechanisms for hydrogen production in purple non-sulfur (PNS) bacteria under temperature stress.

## 1. Introduction

The increasing demand for energy leads to search for alternative energy sources. Hydrogen is a renewable and environmentally safe alternative to fossil fuels, which cause global warming. The ability to employ microorganisms that can use of a variety of renewable substrates make biological hydrogen production a primary choice for the generation of hydrogen.

Purple non-sulfur (PNS) bacteria can produce hydrogen from the degradation of organic substrates under an anaerobic atmosphere and nitrogen limiting conditions using light energy via the nitrogenase enzyme [1,2]. The Gram-negative PNS bacterium *R. capsulatus* has long been studied for its versatile metabolism, nitrogen fixation and hydrogen production. Moreover, this bacterium is suitable for biochemical and genetic approaches since it can be easily mutated by standard procedures [3].

Since hydrogen production in PNS bacteria is catalyzed by nitrogenase, and thus an enzymatic reaction, it is affected by temperature. The optimum temperature range for hydrogen production by *Rhodobacter* species is between 31–36 °C [4]. Accordingly, Androga *et al.* [5] determined that the highest hydrogen productivity of *R. capsulatus* was at 30 °C. Furthermore, the expression of nitrogenase activator *nifA* gene decreased compared to 30 °C when the temperature was 20 and 38 °C [6]. Özgür *et al.* [7] studied the effect of fluctuating temperatures on hydrogen production of an *R. capsulatus hup^−^* mutant in outdoor photobioreactors. They showed a decrease in hydrogen production in daily fluctuations of temperature (15–40 °C) and light/dark cycle. Lack of temperature and light intensity control are the major challenges for the operation of photobioreactors in outdoor conditions. Androga *et al.* [8] investigated the factors affecting hydrogen production of *R. capsulatus hup^−^* strain under outdoor conditions and showed the decrease in hydrogen yield during the winter (October–December) in Ankara, Turkey where night temperatures were less than 5 °C. Avcioğlu *et al.* [9] reported that the temperature in the four-liter outdoor photobioreactor could increase above 45 °C, causing decrease of hydrogen productivity and yield. They concluded that hydrogen production under natural sunlight was affected greatly by seasonal variations in temperature and suggested that the temperature should not exceed 38 °C in outdoor photobioreactors.

Temperature extremes in outdoor photobioreactor operations may cause stress on the microorganism damaging membrane components, proteins and nucleic acids; which may result in lag phase increase, growth rate reduction, or even death [10].

Understanding genetic regulations under temperature stress may help to genetically engineer the bacteria to increase hydrogen yield in large-scale industrial photobioreactors. DNA microarrays are powerful tools for genome-wide expression analysis. Microarray analysis provides a detailed insight into cellular processes by simultaneously monitoring expression levels of thousands of genes [11]. In this study, a custom designed Affymetrix GeneChip^®^. TR_RCH2a520699F (GEO accession number GPL1860) for *R. capsulatus* DSM1710 was constructed. The effects of temperature stress on the hydrogen production metabolism of *R. capsulatus* were investigated at the transcriptome level and reported here for the first time.

## 2. Results and Discussion

### 2.1. Physiological Analyses

**Figure 1:**
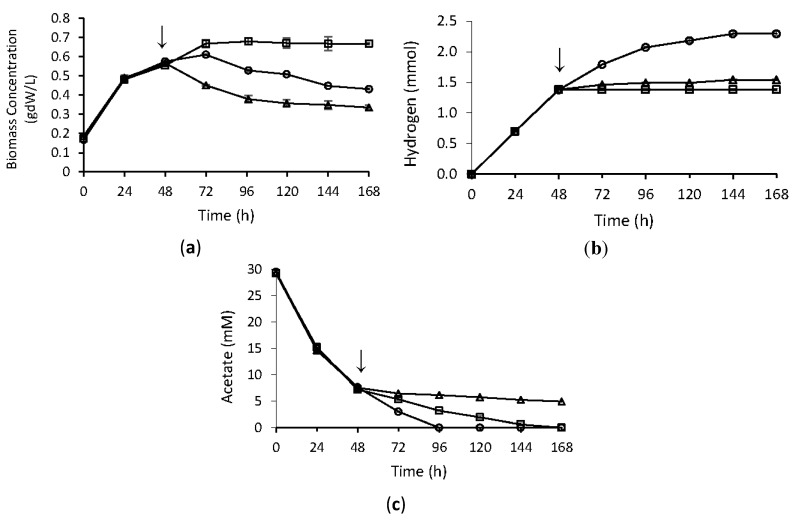
Bacterial growth (**a**); hydrogen production (**b**); and acetate consumption (**c**) of *R. capsulatus* under temperature stress. Arrow indicates the time of stress application. Experiments were carried out as duplicates and error bars indicate ± SEM. (○: control; □: cold stress; Δ: heat stress).

In this study we investigated the effects of temperature stress on hydrogen production metabolism of *R. capsulatus.* The bacteria were grown under anaerobic conditions in hydrogen production medium at 30 °C for 48 h. After 48 h, when the hydrogen production was at exponential phase, bacteria received either cold stress at 4 °C, or heat stress at 42 °C. Physiological data on H_2_ production, growth and substrate consumption were followed for five days after heat and cold stress were applied. Control groups were kept at 30 °C throughout the experiment. As shown in Figure 1, until stress application, growth, hydrogen production, and substrate consumption profiles were very similar to each other. Hydrogen production immediately stopped upon both cold and heat stress applications. Heat stress causes the death of the bacteria in the long term, however, bacteria continued to survive under cold stress. Substrate uptake rate decreased under cold stress treatment, but all acetate was consumed. On the other hand, upon heat stress, treatment uptake of acetate ceased completely.

### 2.2. Microarray Analysis

**Figure 2:**
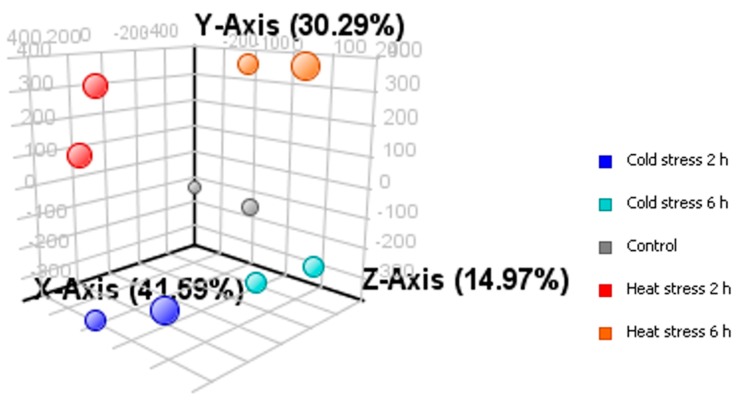
Principal Component Analysis of duplicate samples. The percentages of the total variation that are accounted by the 1st, 2nd, and 3rd principal components are shown on the *x*, *y* and *z* axes labels.

Transcriptome analysis was carried out using samples taken at the 2nd and 6th h of stress applications. Microarray data quality was checked as previously described, and data analysis was continued with the chips that met comparable quality control parameters [12]. In order to explore the correlations between duplicate samples, Principal Component Analysis was performed. Duplicate samples were close to each other and clearly separated from the rest (Figure 2). Moreover, the Pearson correlation coefficients between the duplicates were significant (Table 1). Together with physiological analyses, these results reveal that the differences in gene expression levels are a result of temperature stress application. According to the Microarray Quality Control (MAQC) Project [13], significance analysis was based on a fold change ≥2.0 with the *p*-value cut-off at 0.1.

**Table 1:** Correlation coefficients of duplicate samples of temperature treatments.

Samples	Correlation Coefficients between Duplicates
Control	0.975
Cold stress 2 h	0.959
Cold stress 6 h	0.985
Heat stress 2 h	0.967
Heat stress 6 h	0.987

To test the validity of the microarray results we performed real-time qPCR analysis on three genes: *pufM*, *atpF* and *oppA*. These genes were chosen from three different metabolic pathways: photosynthesis, electron transport system, and membrane transport. The *pufM* gene encodes the medium subunit of the photosynthetic reaction center. It was significantly down regulated (3.1-fold) at 6 h of heat stress. The product of *atpF* is B subunit of ATP synthase F_0_ and the product of *oppA* is an oligopeptide ABC transporter. *atpF* was up regulated 3.57-fold at 2 h of cold stress; *oppA* was down regulated 3.2-fold at 2 h of heat stress. Table 2 shows the corroboration of our microarray results with the real-time qPCR analysis, which provides an independent verification of transcript level changes.

**Table 2:** Transcript level comparison genes of *R. capsulatus* between real-time qPCR and microarray analyses.

Gene	Transcript Change with Real-Time qPCR	Transcript Level Change with Microarray	Forward Primer (5′>3′)	Reverse Primer (3′>5′)
16S rRNA	1.0	1.0	GCTAGTAATCGCGTAACAGCA	CAGTCACTGAGCCTACCGT
*atpF*	+2.50 ± 0.23	+3.57	ACGTTCCTGCTTGTTGCTCT	TCGAGGGAACCTTGAACTTG
*pufM*	−1.43 ± 0.02	−3.10	CACCATCGGTGTGTGGTACT	AGACACCACCCTGTTTCAGC
*oppA*	−2.58 ± 0.12	−3.20	AGGAACTGCTCAAACCGATG	GTCCTTGTAGTCCGCAAACC

We found that expressions of 328 genes out of 4052 probe sets changed significantly under cold stress, and 293 genes under heat stress.

**Figure 3:**
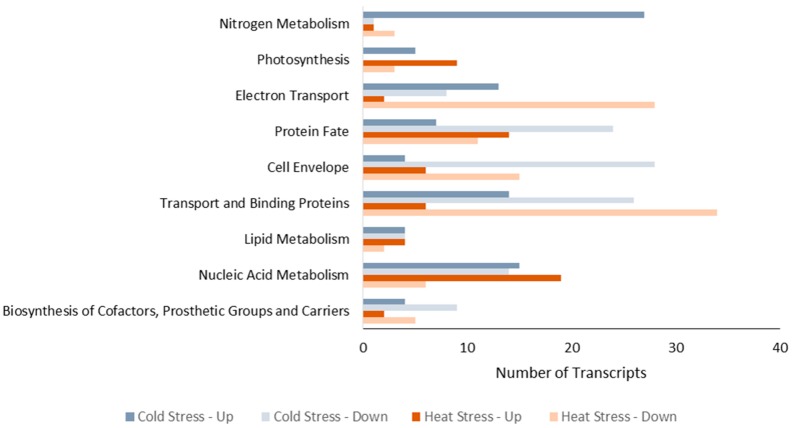
Metabolic distributions of genes with significant change in expression levels under cold and heat stress. Data are given as the number of transcripts.

We grouped the genes into appropriate metabolisms using information from the web site of the *R. capsulatus* genome (http://onco.img.cas.cz/rhodo/results/index.html) and the protein grouping of Onder *et al.* [14]. Figure 3 shows the metabolic distribution of significantly changed genes under both stress conditions. The distribution of genes in nitrogen metabolism, photosynthesis, and electron transport differ significantly between cold and heat stress. These are the most important metabolisms for hydrogen production since photoproduction of hydrogen in PNS bacteria is primarily related to nitrogen metabolism, together with electron transport and photosynthesis [15]. The detailed lists of expression levels of significantly changed genes are given in Table 3 and Table 4

**Table 3:** Differentially expressed *R. capsulatus* genes under cold stress.

Functional Group	Probe Set ID	Gene Symbol	Description	Fold Change		*p* Value
				2 h	6 h	
Nitrogen Metabolism	RCAP_rcc00566_s_at	*nifB*	Nitrogenase cofactor biosynthesis protein NifB	+6.317	+2.487	0.0204
	RCAP_rcc00571_at	*nifD*	Nitrogenase molybdenum-iron protein α chain	+59.112	+38.480	0.0176
	RCAP_rcc03280_at	*nifE*	Nitrogenase molybdenum-iron cofactor biosynts protein NifE	+3.069	+1.223	0.0089
	RCAP_rcc00572_at	*nifH*	Nitrogenase iron protein	+99.953	+44.111	0.006
	RCAP_rcc00570_at	*nifK*	Nitrogenase molybdenum-iron protein β chain	+29.376	+16.530	0.003
	RCAP_rcc03279_at	*nifN*	Nitrogenase molybdenum-iron cofactor biosynthesis protein NifN	+6.598	+1.957	0.0038
	RCAP_rcc00586_at	*anfD*	Nitrogenase iron-iron protein, α subunit	+23.486	+13.126	0.0301
	RCAP_rcc00587_at	*anfG*	Nitrogenase iron-iron protein, delta subunit	+37.180	+22.194	0.0379
	RCAP_rcc00585_at	*anfH*	Nitrogenase iron protein	+33.189	+20.210	0.0873
	RCAP_rcc01674_at	*glnA*	Glutamine synthetase	+2.249	+2.212	0.0027
	RCAP_rcc01673_at	*glnB*	Nitrogen regulatory protein P-II	+2.756	+2.093	0.0355
Photosynthesis	RCAP_rcc01830_at	*fba*	Fructose-bisphosphate aldolase	+2.056	+3.441	0.0137
	RCAP_rcc01834_at	*fbp*	Fructose-bisphosphatase	+2.748	+2.564	0.0988
	RCAP_rcc00744_at	*atpF*	ATP synthase F_0_, B subunit	+3.575	+1.640	0.0535
Electron Transport	RCAP_rcc03284_at	*fdxN*	Ferredoxin I	+23.668	+10.214	0.0093
	RCAP_rcc03285_at	*fdxC*	Ferredoxin IV	+13.582	+2.455	0.0000
	RCAP_rcc03287_at	*rnfA*	Electron transport complex protein RnfA	+20.417	+5.874	0.0033
	RCAP_rcc03288_at	*rnfB*	Electron transport complex protein RnfB	+9.373	+1.972	0.0018
	RCAP_rcc03289_at	*rnfC*	Electron transport complex protein RnfC	+17.068	+7.526	0.0036
Electron Transport	RCAP_rcc03290_at	*rnfD*	Electron transport complex protein RnfD	+7.219	+4.282	0.0042
	RCAP_rcc03292_at	*rnfE*	Electron transport complex protein RnfE	+7.124	+5.601	0.0039
	RCAP_rcc03291_at	*rnfG*	Electron transport complex protein RnfG	+8.091	+3.526	0.0028
	RCAP_rcc00768_at	*hupB*	Hydrogenase, large subunit	+1.442	+3.407	0.0749
Protein Fate	RCAP_rcc00223_at	*dnaJ*	Chaperone DnaJ	−4.573	−2.022	0.0422
	RCAP_rcc00224_at	*dnaK*	Chaperone DnaK	−5.482	−1.307	0.0031
	RCAP_rcc02977_at	*clpA*	ATP-dependent Clp protease, ATP-binding subunit ClpA	−3.889	−1.271	0.0850
	RCAP_rcc03406_at	*clpB*	Chaperone ClpB	−12.622	−1.735	0.0035
	RCAP_rcc02609_at	*clpP*	ATP-dependent Clp protease, ATP-binding subunit ClpX	−2.995	−1.054	0.0292
	RCAP_rcc01167_at	*clpS*	ATP-dependent Clp protease adaptor protein ClpS	−3.602	−1.333	0.0108
	RCAP_rcc02478_at	*groL*	Chaperonin GroL	−8.799	−1.019	0.0037
	RCAP_rcc02477_at	*groS*	Chaperonin GroS	−6.357	−1.041	0.0021
	RCAP_rcc02818_at	*ibpA*	Small heat shock protein IbpA	−13.62	−1.762	0.0023
	RCAP_rcc02583_at	*lon*	ATP-dependent protease La	−5.656	−1.884	0.0193
	RCAP_rcc00034_s_at	*hslU*	ATP-dependent hsl protease ATP-binding subunit hslU	−5.356	−3.532	0.0979
	RCAP_rcc00035_s_at	*hslV*	ATP-dependent protease HslV	−5.836	−2.666	0.0585
	RCAP_rcc00480_at	*rpsU*	30S ribosomal protein S21	+5.586	+2.147	0.0835
	RCAP_rcc00327_at	*rplQ*	50S ribosomal protein L17	+4.036	+2.120	0.0982
Cell Envelope Biogenesis	RCAP_rcc00373_at	*mdoH*	Glucans biosynthesis glucosyltransferase H	−4.527	−1.843	0.0506
	RCAP_rcc01872_at	*lpxD*	UDP-3-*O*-[3-hydroxymyristoyl] glucosamine *N*-acyltransferase	−2.652	−1.499	0.0668
	RCAP_rcc03179_at	*phbB*	Acetoacetyl-CoA reductase	+2.664	+1.002	0.0081
Nucleic Acid Metabolism	RCAP_rcc00561_at	*mopA*	Molybdenum transport operon repressor MopA	+2.673	+1.150	0.0027
	RCAP_rcc00568_at	*rpoN*	RNA polymerase σ-54 factor	+6.031	+2.602	0.0269
	RCAP_rcc01801_at	*hfq*	RNA chaperone Hfq	+2.452	−1.020	0.0017
	RCAP_rcc02165_at	*rne*	Ribonuclease E	+3.679	−1.004	0.0014
	RCAP_rcc01384_at	*uvrB*	UvrABC system protein B	−4.826	−1.998	0.0136
	RCAP_rcc00201_at	*-*	site specific DNA methyltransferase	+2.578	+1.070	0.0041
Transporter and Binding Proteins	RCAP_rcc00563_at	*modB*	Molybdenum ABC transporter, permease protein ModB	+3.113	+1.062	0.0033

**Table 4:** Differentially expressed *R. capsulatus* genes under heat stress.

Functional Group	Probe Set ID	Gene Symbol	Description	Fold Change		*p* Value
				2 h	6 h	
Nitrogen Metabolism	RCAP_rcc01674_at	*glnA*	Glutamine synthetase	−5.476	−9.266	0.0013
	RCAP_rcc03387_at	*glnB*	Nitrogen regulatory protein P-II	−2.532	−7.445	0.0912
Photosynthesis	RCAP_rcc00733_at	*sdhA*	Succinate dehydrogenase, flavoprotein subunit	−1.982	−2.147	0.0089
	RCAP_rcc00736_at	*sdhB*	Succinate dehydrogenase, iron-sulfur subunit	−2.558	−3.508	0.0324
	RCAP_rcc02531_at	*pucA*	Light-harvesting protein B-800/850, α chain	+1.070	−2.053	0.0405
	RCAP_rcc00660_at	*pucC*	PucC protein	−2.221	−1.728	0.0855
	RCAP_rcc00693_at	*pufL*	Photosynthetic reaction center, L subunit	−1.894	−3.864	0.0825
	RCAP_rcc00694_at	*pufM*	Photosynthetic reaction center, M subunit	−2.156	−3.102	0.0691
	RCAP_rcc02970_at	*atpC*	ATP synthase F1, epsilon subunit	−1.981	−2.559	0.0380
	RCAP_rcc02971_at	*atpD*	ATP synthase F1, β subunit	−6.198	−9.121	0.0211
	RCAP_rcc02150_at	*acpA*	Aconitate hydratase	−2.482	−2.801	0.0930
Electron Transport	RCAP_rcc00573_at	*fdxD*	Ferredoxin V	−3.345	−2.949	0.0075
	RCAP_rcc00767_at	*hupA*	Hydrogenase, small subunit	−2.436	−3.333	0.0778
	RCAP_rcc00768_at	*hupB*	Hydrogenase, large subunit	−3.744	−5.254	0.0249
	RCAP_rcc00769_at	*hupC*	Hydrogenase, cytochrome b subunit	−2.401	−2.354	0.0387
	RCAP_rcc01517_at	*nuoA*	NADH-quinone oxidoreductase, A subunit	−2.363	−2.214	0.0795
	RCAP_rcc01518_at	*nuoB*	NADH-quinone oxidoreductase, B subunit	−2.401	−2.791	0.0719
	RCAP_rcc01519_at	*nuoC*	NADH-quinone oxidoreductase, C subunit	−2.884	−2.758	0.0153
	RCAP_rcc01520_at	*nuoD*	NADH-quinone oxidoreductase, D subunit	−3,101	−4.132	0.0043
	RCAP_rcc01521_at	*nuoE*	NADH-quinone oxidoreductase, E subunit	−2,700	−2.869	0.0289
	RCAP_rcc01527_at	*nuoG*	NADH-quinone oxidoreductase, G subunit	−1,720	−2.262	0.0553
	RCAP_rcc01529_at	*nuoH*	NADH-quinone oxidoreductase, H subunit	−2.118	−1,663	0.0749
Protein Fate	RCAP_rcc03406_at	*clpB*	Chaperone ClpB	−2.502	−2.580	0.0120
	RCAP_rcc02609_at	*clpP*	ATP-dependent Clp protease, ATP-binding subunit ClpX	−2,358	−3.859	0.0676
	RCAP_rcc01167_at	*clpS*	ATP-dependent Clp protease adaptor protein ClpS	+1291	+2.704	0.0839
	RCAP_rcc02583_at	*Lon*	ATP-dependent protease La	−4.331	−2.544	0.0235
	RCAP_rcc00480_at	*rpsU*	30S ribosomal protein S21	+12.697	+4.962	0.0026
	RCAP_rcc00321_at	*rplO*	50S ribosomal protein L15	+8.431	+8.222	5.4674 × 10^−4^
	RCAP_rcc00361_at	*rpmE*	50S ribosomal protein L31	+3.899	+2.197	0.0983
Cell Envelope Biogenesis	RCAP_rcc00350_at	*rpmH*	50S ribosomal protein L34	+4.232	+3.551	0.0134
	RCAP_rcc02380_at	*ftsI*	Peptidoglycan synthetase FtsI	−2.279	−2.546	0.0851
	RCAP_rcc01678_at	*acpP*	Acyl carrier protein	+4.132	+1.487	0.0863
	RCAP_rcc00559_at	*pmtA*	Phosphatidylethanolamine *N*-methyltransferase	+3.117	+2.273	0.0809
	RCAP_rcc00028_at	*Idi*	Isopentenyl-diphosphate delta-isomerase	+4.085	+2.981	0.0973
Nucleic Acid Metabolism	RCAP_rcc00349_at	*rnpA*	Ribonuclease P	+2.065	+1.919	0.0393	
	RCAP_rcc00286_at	*nusG*	Transcription antitermination protein NusG	+3.113	+2.461	0.0678	
	RCAP_rcc03054_at	*rpoD*	RNA polymerase σ factor RpoD	−2.919	−2.406	0.0329	
	RCAP_rcc00458_at	*rpoH*	RNA polymerase σ-32 factor	−3.361	−1.667	0.0032	
	RCAP_rcc01751_at	*recA*	RecA protein	+1.895	+2.357	0.0845	
	RCAP_rcc00201_at	*-*	Site specific DNA methyltransferase	+2.231	−1.070	0.0013	
Transporter and Binding Proteins	RCAP_rcc01243_at	*potA*	Polyamine ABC transporter, ATP binding protein PotA	−8.289	−10.273	0.0073	
	RCAP_rcc01245_at	*potB*	Polyamine ABC transporter, permease protein PotB	−4.566	−7.072	0.0040	
	RCAP_rcc01244_at	*potD*	Polyamine ABC transporter, periplasmic polyamine-binding protein PotD	−14.108	−15.842	0.0014	
	RCAP_rcc02186_at	*potF*	Spermidine/putrescine ABC transporter, periplasmic spermidine/putrescine-binding protein PotF	−4.010	−4.508	0.0193	
	RCAP_rcc02183_at	*potG*	Spermidine/putrescine ABC transporter, ATP-binding protein PotG	−3.135	−2.833	0.0203	
	RCAP_rcc01895_at	*potH*	Spermidine/putrescine ABC transporter, permease protein	−2.976	−2.301	0.0588	
	RCAP_rcc01246_at	*potI*	Polyamine ABC transporter, permease protein PotI	−3.940	−4.112	0.0304	
	RCAP_rcc01879_at	*sufC*	FeS assembly ATPase SufC	−6.598	−6.351	2.1582 × 10^−4^	
	RCAP_rcc00092_at	*feoB*	Ferrous iron transport protein B	−2.362	−1.524	0.0338	
	RCAP_rcc01878_at	*sufD*	FeS assembly protein SufD	−4.363	−4.224	0.0306	
	RCAP_rcc01376_at	*dctP*	TRAP dicarboxylate transporter, DctP subunit	−1.538	−2.436	0.0186	
	RCAP_rcc00706_at	*oppA*	Oligopeptide ABC transporter, Periplasmic oligopeptide-binding protein OppA	−3.203	−3.085	0.0069

### 2.3. Effect of Temperature Stress on Hydrogen Production Metabolism of R. capsulatus

Photofermentative hydrogen production by *R. capsulatus* is governed mostly by photosynthesis, electron transport, and nitrogen metabolism [15]. These were greatly affected by temperature stress.

Under cold stress, overall increase of gene expression of nitrogen metabolism and the electron transport system is remarkable. The nitrogenase enzyme genes, *nif* and *anf*, were highly up regulated (Table 3). The nitrogenase enzyme is primarily responsible for nitrogen fixation; however one mole of hydrogen is also produced during the following process:

N_2_ + 8H^+^ + 8e^−^ + 16ATP → 2NH_3_ + H_2_ + 16ADP + 16P_i_ (1)

Under nitrogen limiting conditions, nitrogenase takes up the electron from ferredoxin/flavodoxin and reduces the protons to molecular hydrogen via the following reaction:

2H + 2e^−^ + 4ATP → H_2_ + 4ADP + 4P_i_ (2)

Nitrogenase acts as a redox balancing system and reduces the electrons coming from cyclic photosynthesis and oxidation of organic acids [16,17]. The genes encoding ferredoxins (*fdxC* and *fdxN*), which carry the electrons to the nitrogenase from the electron transfer chain in the membrane, were also significantly up regulated (13–23-fold). Similarly, electron transport complex genes (*rnf* genes), which serve as electron donors to nitrogenase under photosynthetic conditions [18] were up regulated up to 20-fold. The molecular hydrogen produced by nitrogenase is recycled by the hydrogenase enzyme encoded by *hup* genes. Under cold stress, hydrogenase large subunit gene *hupB* was up regulated by three fold, which is consistent with up regulation of nitrogenase genes. The expression of ATP synthase (ATPase) F_0_ subunit gene (*atpF*) was up regulated 3.5-fold under cold stress. This finding is also consistent with up regulation of nitrogenase genes. This indicates that nitrogenase and electron transport metabolism are co-regulated under cold stress.

The regulators of nitrogen metabolism, *glnA* and *glnB*, were also up regulated under cold stress. Moreover, the alternative σ factor gene *rpoN* was up regulated six fold under cold stress. Alternative σ factors control transcription of specific regulons during special physiological conditions, such as stress. Several alternative σ factors mediating different stress responses have been defined in Bacteria. Among them, RpoN, σ 54, is an activator of *nif* genes [19]. Its up regulation under cold stress is consistent with the up regulation of nitrogenase genes under cold stress.

Although the nitrogenase genes were highly up regulated, hydrogen production stopped after application of cold stress, as shown in Figure 1. This result shows that there is an apparent increase in mRNA levels of *nif* genes at the time of sampling for microarray analysis (2 and 6 h of stress). However, the transcripts may not have been translated into functional proteins, or the activity of the nitrogenase enzyme could have been controlled at the post-translational level. Further studies are required both at the translational and enzyme activity level in order to clarify this point.

Under heat stress, on the other hand, no significant change in the expression of nitrogenase genes was observed, while electron transport system genes and photosynthesis genes were down regulated (Table 4). The down regulated genes include ATP synthase subunit genes (*atpC* and *atpD*), hydrogenase (*hupA*, *hupB*, *hupC*), NADH quinone oxidoreductase genes (*nuoA*, *nuoB*, *nuoC*, *nuoD*, *nuoE*, *nuoG*, *nuoH*), and photosystem reaction center genes (*puf* and *puc* genes). NADH quinone oxidoreductase links electrons from ubiquinone, resulting in the generation of a proton gradient for ATP synthesis [20]. The down regulation of these genes suggests impairment in energy production of *R. capsulatus* by heat stress. These results are also consistent with our physiological results demonstrating the death of bacteria under heat stress, which might be due to energy impairment (Figure 1).

### 2.4. Effect of Temperature Stress on Protein Metabolism of R. capsulatus

The ribosomal protein genes (both 30S and 50S) were up regulated under both stress conditions. Macario and Macario [21] suggested that a higher transcription rate of a gene and increased life span of its mRNA are necessary to cope with heat stress. Increased temperature induces protein degradation, thus under heat stress cells require higher ribosomal protein concentration to synthesize necessary proteins. On the other hand, the transcription rate decreases with low temperature and the tendency to restore transcription levels explains the up regulation under cold stress. The heat shock protein (HSP) genes *dnaJ*, *dnaK*, *groEL*, *groES*, and *clp* encode molecular chaperons and proteases, which respectively mediate the correct folding and assembly of proteins, and degrade abnormal proteins [22]. These proteins are synthesized under non-stress conditions at lower rates, but their expression increases rapidly and transiently under heat shock to cope with increased damaged proteins [23]. In this study, the change in expression values of HSPs after heat stress application was found to be insignificant. This is because the fast and transient induction of HSP genes was missed in our experimental design. Cold stress application caused a significant decrease in expression of HSP genes (*dnaJ*, *dnaK*, *clpA*, *clpB*, *clpP*, *clpS*, *groL*, *groS*, *ibpA*, *lon*, *hslU*, *hslV*) compared to non-stress conditions. Cold stress treatment also decreased the transcription and translation rates. Therefore, HSP genes were down regulated to protect the present proteins from degradation. It is known that under cold stress, bacteria can save valuable resources by down regulation of HSP genes [24].

### 2.5. Effect of Temperature Stress on Lipid, Cell Envelope, and Trasporter Metabolisms of R. capsulatus

The membrane is vital for selective transport, the growth and survival of bacteria. The membrane lipid composition changes to maintain membrane fluidity at lower temperature which increases under heat stress, and decreases under cold stress [25]. Our microarray analysis suggests that the expression of genes involved in the biosynthesis of the cell envelope and cell membrane were up regulated under heat stress. The *pmtA* gene, which is crucial for formation of photosynthetic membrane in PNS bacteria [26], was increased three fold. Isopentenyl-diphosphate delta-isomerase gene *idi* was also up regulated (four fold) under heat stress. This enzyme is the last enzyme in the non-mevalonate pathway, which contributes to the biosynthesis of carotenoids and bacteriochlorophylls, which are important members of the photosynthetic center required to build the photosynthetic membrane system [27]. The acyl carrier protein gene *acpP*, which plays a significant role in fatty acid biosynthesis [28], was also up regulated. These results suggest that heat stress results in damage to the membrane structure and integrity.

Another effect of heat stress was the 4.5–15-fold decrease in the expressions of polyamine and spermidine/putrescine ABC transporter genes. Polyamines stimulate RNA and protein synthesis, and thus are required for growth [29]. Fe/S assembly genes *sufC* and *sufD*, which support biosynthesis of the Fe/S clusters in photosystem and electron transport [30] were 4–6-fold down regulated under heat stress. Consistently, expression of ferrous iron transporter B gene *feoB* was also down regulated under heat stress. These down regulations are also consistent with decreased electron transport genes under heat stress.

Under cold stress, the molybdenum ABC transporter gene *modB* was up regulated. Molybdenum is present in the FeMo cofactor of nitrogenase MoFe protein. Thus the increase in nitrogenase expression brought about an increased need for molybdenum. The expression of genes taking place in biosynthesis of the cell envelope were down regulated after two hours of cold stress application, but returned to control levels by six hours. This suggests that the bacteria adapt to cold stress.

### 2.6. Effect of Temperature Stress on Nucleic Acid Metabolism of R. capsulatus

The other important pathway for the bacteria to cope with stress and to survive is nucleic acid metabolism. Temperature stress can affect the efficiency of transcription, translation and recombination. Both cold and heat stresses up regulated the DNA methyltransferase gene, whose product methylates DNA and protects it from degradation. Heat stress also up regulated the *recA* gene which plays a role in DNA damage repair by recombination. Ribonuclease E was up regulated under cold stress together with that of RNA chaperone Hfq and polynucleotidyltransferase. Hfq protein assists RNA-RNA interactions and affects the translation of specific transcripts, and contributes to complex post-transcriptional networks [31]. In *R. sphaeroides*, Hfq supports small RNA (sRNA) function under ^1^O_2_ stress [32]. Increased expression of Hfq (2.4-fold) may indicate that noncoding RNAs may play a role in the stress response.

## 3. Experimental Section

### 3.1. Growth of Bacteria and Stress Treatments

The purple non-sulfur bacterium *Rhodobacter capsulatus* strain DSM1710 was obtained from DSMZ (German Collection of Microorganisms and Cell Cultures, Braunschweig, Germany). The bacteria were grown photoheterotrophically on a modified medium of Biebl and Pfennig [33] containing 20 mM acetate and 10 mM glutamate as carbon and nitrogen sources, respectively, at 30 °C under a continuous illumination of 114 W/m^2^ with 100 W tungsten lamps in batch mode. From the grown bacterial culture, inoculations to hydrogen production medium (30 mM acetate/2 mM glutamate) were made under aseptic conditions to provide a starting bacterial concentration of OD_660nm_ = 0.230–0.250 (0.127–0.137 g dry cell weight) in 50 mL of total culture volume in 55 mL of glass reactor bottles. The cultures were sparged with argon gas to generate an anerobic environment, connected to collection tubes filled with distilled water and the produced gas was collected by water displacement method [34] and incubated at 30 °C for 48 h. After 48 h, two photobioreactors were kept at 30 °C to be used as control cultures, two of them were transferred to an incubator that was set to 4 °C and two were transferred to an incubator at 42 °C. Samplings were done periodically. Bacterial growth was followed by measuring the OD at 660 nm by spectrophotometer (Shimadzu UV-1201, Kyoto, Japan). The regression coefficient (R^2^) of the optical density (OD_660nm_) *vs.* dry cell weight calibration curve is 0.55, therefore the OD_660nm_ values were multiplied with 0.55 to obtain the dry cell weight/L values. The gas samples were taken from the top of the reactor bottles by a gas tight syringe (Hamilton, 22 GA 500 µL-No. 1750, Bonaduz, Switzerland). The composition of 100 µL gas samples from the top of the reactor bottles was analyzed by gas chromatography (Agilent Technologies 6890N, Santa Clara, CA, USA) with the column SupelcoCarboxen 1010 (Sigma-Aldrich, St. Louis, MO, USA). The carrier gas was argon with a flow rate of 26 mL/min. The oven temperature was 140 °C and the temperatures of injector and detector were 160 and 170 °C, respectively. The moles of the produced hydrogen were calculated by using ideal gas equation (PV = nRT). The software used was Agilent Chemstation ver.B.01.01 (Agilent Technologies, Santa Clara, CA, USA). High Performance Liquid Chromatography (HPLC) equipped with an Alltech IOA-1000 (300 mm × 7.8 mm, Alltech, Deerfield, IL, USA) column was used to analyze the acetate consumption. Ten microliters of sample were injected into the system by the autosampler (Shimadzu SIL-20AC, Kyoto, Japan). The UV detector (Shimadzu SPD-20A, Kyoto, Japan) detected the organic acids at 210 nm wavelength. The oven temperature was kept at 66 °C. The mobile phase was 0.085 M H_2_SO_4_, with a flow rate of 0.4 mL/min with a low gradient pump (Schimadzu LC-20AT, Kyoto, Japan).

### 3.2. Microarray Analysis

Since a microarray chip for *R. capsulatus* was not available, a 100-3660 format GeneChip^®^ Custom array having 4052 probe sets for 3685 open reading frames was designed by our group according to the GeneChip^®^ Custom Expression Array Design Guide (Affymetrix, Santa Clara, CA, USA). The whole genome sequence of *R. capsulatus* was obtained from http://rhodo.img.cas.cz [35].

The total RNA was isolated from 1 × 10^9^ cells/mL bacterial samples drawn at the second and sixth hours of stress treatments. TRIzol Reagent (Invitrogen, Carlsbad, CA, USA) was used to isolate RNA. The yield and RNA purity were determined spectrophotometrically by using NanoDrop 2000 (Thermo Scientific, Waltham, MA, USA) and integrity of RNA was checked using Agilent Bioanalyzer (Agilent Technologies, Santa Clara, CA, USA).

cDNA of the total RNA was produced by reverse transcription reaction according to the Affymetrix Expression Analysis Technical Manual for Prokaryotic Target Preparation Manual. cDNA was purified with MinElute PCR Purification columns (QIAGEN, Hilden, Germany). It was then fragmented, labeled with biotin and hybridized onto the array according to the manufacturer’s instructions. GeneChip^®^ Scanner 3000 was employed for scanning the hybridized arrays. Gene Chip Operating Software (Affymetrix, Santa Clara, CA, USA) was used for the feature extraction, image analysis and data extraction process. The RMA normalization was performed using GeneSpring GX version 11 (Agilent Technologies, Santa Clara, CA, USA). After that probe sets were filtered by expression based on raw intensity values with a lower percentile cut off of 20. One-way Analysis of Variance (ANOVA) was used to calculate the *p* values and *p* value correction was performed by Benjamini Hochberg method. The genes with satisfying *p* values (*p* ≤ 0.1) were further filtered by fold change analysis with the 2.0-fold change cut-off.

The data is available in Gene Expression Omnibus with the accession number GSE53477.

### 3.3. Real Time PCR Analysis

In order to determine the validity of the microarray results, transcript level changes were compared between microarray and real time PCR data. Genes and primer sequences are listed in Table 2. The housekeeping gene 16S rRNA was used as endogenous control. Roche LightCycler 1.5 (Roche GmbH, Mannheim, Germany) was used for the reactions, the reaction mix was LightCycler FastStart DNA Master SYBR Green I. For each gene two biological replicates with three technical replicates were employed. Reaction mixtures were first incubated at 95 °C for 10 min, followed by 40 cycles of denaturation, annealing and extension reactions which lasted 10 s at 95 °C, 5 s at 60 °C and 5 s at 72 °C, respectively. Melt curve analysis was performed to evaluate PCR specificity, and resulted in primer–specific melting temperatures. PCR efficiencies were derived from standard curve slopes in the LightCycler Software 4.0 (Roche, Mannheim, Germany). In this study, transcript level changes are reported as relative quantification based on relative expression of the target gene *vs.* 16S rRNA gene.

## 4. Conclusions

In this study whole genome DNA microarray analysis was utilized to evaluate global gene expression of *Rhodobacter capsulatus* in response to temperature stress. This is the first study that characterizes temperature stress effects on the hydrogen production metabolism of *R. capsulatus* at the whole genome transcriptional level. Expression profiles indicate energy impairment with elevated temperatures. Heat stress repressed the expression of photosynthesis, electron transport system, tricarboxylic acid (TCA) cycle and ATPase genes. This energy impairment limits bacterial growth and causes death under heat stress. In contrast, energy metabolism related genes such as electron transport complex protein and ATPase genes were induced by cold stress and the cell concentration was maintained.

Under heat stress, the genes for biosynthesis of fatty acids and phospholipids were up regulated. These groups of genes were down regulated after two hours of cold stress application but returned to the expression levels of control condition after six hours. This suggests that the bacteria can adjust their metabolism for adaptation to cold stress.

Nitrogen metabolism genes, especially nitrogenase genes, were highly up regulated under cold stress, which is a novel finding. However, nitrogenase gene expression did not significantly change under heat stress. The up regulation of hydrogen production genes while the hydrogen production actually stops should indicate that the bacteria try to compensate the reduced metabolic flow through this pathway on the gene regulatory level. The reason for the reduction may likely be the temperature sensitivity of one or more enzymatic reactions involved.

The results of this study may be helpful for understanding critical points that should be considered in genetic manipulations of PNS bacteria for improvement of H_2_ production capacity under temperature stress.
